# Optimising personal continuity for older patients in general practice: a study protocol for a cluster randomised stepped wedge pragmatic trial

**DOI:** 10.1186/s12875-021-01511-y

**Published:** 2021-10-20

**Authors:** Lex J. J. Groot, Henk J. Schers, Jako S. Burgers, Francois G. Schellevis, Martin Smalbrugge, Annemarie A. Uijen, Peter M. van de Ven, Henriëtte E. van der Horst, Otto R. Maarsingh

**Affiliations:** 1 Department of General Practice, Amsterdam University Medical Centre, location VU University Medical Centre, van der Boechorststraat 7, 1081 BT Amsterdam, the Netherlands; 2 Department of Primary and Community Care, Radboud University Nijmegen Medical Centre, Geert Grooteplein 21, 6525 EZ Nijmegen, The Netherlands; 3 MUMC+/ Maastricht University, Department of General Practice, Care and Public Health Research Institute (CAPHRI), Universiteitssingel 40, 6229 ER Maastricht, the Netherlands; 4 Department of Medicine for Older People, Amsterdam University Medical Centre, location VU University Medical Centre, De Boelelaan 1109, 1081 HV Amsterdam, the Netherlands; 5 Department of Epidemiology and Data Science, Vrije Universiteit Amsterdam, De Boelelaan 1089a, 1081 HV Amsterdam, the Netherlands

**Keywords:** Continuity of patient care, Aged, Intervention, Quality improvement

## Abstract

**Background:**

Continuity of care, in particular personal continuity, is a core principle of general practice and is associated with many benefits such as a better patient-provider relationship and lower mortality. However, personal continuity is under pressure due to changes in society and healthcare. This affects older patients more than younger patients. As the number of older patients will double the coming decades, an intervention to optimise personal continuity for this group is highly warranted.

**Methods:**

Following the UK Medical Research Council framework for complex Interventions, we will develop and evaluate an intervention to optimise personal continuity for older patients in general practice. In phase 0, we will perform a literature study to provide the theoretical basis for the intervention. In phase I we will define the components of the intervention by performing surveys and focus groups among patients, general practitioners, practice assistants and practice nurses, concluded by a Delphi study among members of our group. In phase II, we will test and finalise the intervention with input from a pilot study in two general practices. In phase III, we will perform a stepped wedge cluster randomised pragmatic trial. The primary outcome measure is continuity of care from the patients’ perspective, measured by the Nijmegen Continuity Questionnaire. Secondary outcome measures are level of implementation, barriers and facilitators for implementation, acceptability and feasibility of the intervention. In phase IV, we will establish the conditions for large-scale implementation.

**Discussion:**

This is the first study to investigate an intervention for improving personal continuity for older patients in general practice. If proven effective, our intervention will enable General practitioners to improve the quality of care for their increasing population of older patients. The pragmatic design of the study will enable evaluation in real-life conditions, facilitating future implementation.

**Trial registration number:**

Netherlands Trial Register, trial NL8132. Registered 2 November 2019.

**Supplementary Information:**

The online version contains supplementary material available at 10.1186/s12875-021-01511-y.

## Background

Continuity of care is a core principle of general practice [1]. It denotes coherent care that is adjusted to the health needs and personal context of a patient [2, 3]. Continuity of care is defined by the following three dimensions [4]:

1. “Personal continuity: having a personal provider in every separate care setting who knows and follows the patient;
2. Team continuity: exchange of relevant patient information and cooperation between care providers within one care setting to ensure that care is connected;
3. Cross-boundary continuity: exchange of relevant patient information and cooperation between care providers from different settings to ensure that care is connected.“

Personal continuity is highly valued by both patients and general practitioners (GPs) [5–7] and there is a growing body of evidence for its benefits. Personal continuity is associated with higher quality of GP care [8–11], higher medication adherence [11–13], higher uptake of preventive care [11, 14], better patient-provider relationship [15, 16], higher patient and doctor satisfaction [15–18], higher quality of life [19–21], less overuse of medical procedures [22], lower use of hospital services, lower admission rates [13, 17, 23–27], and an overall reduction in healthcare costs [28–30]. Furthermore, several studies found that personal continuity is associated with lower mortality [29–36].

Over the last decades, changes in in society and healthcare have put a strain on the provision of personal continuity [37, 38]. There is an increase of patients with chronic diseases and these patients more often receive care from various healthcare providers from different organisations [3]. As GPs increasingly work part time, organise themselves into larger group practices and are more likely to work as a locum [39–41], it may be more difficult for GPs to build and maintain a personal relationship with their patients. Additionally, some patients tend to prioritise fast access over personal continuity [37, 42], further challenging personal continuity.

Older patients are more likely to have several chronic conditions [43] and are therefore more at risk for fragmentation and discontinuity of care [26, 44, 45]. At the same time, there is evidence that this group in particular benefits from personal continuity [1, 13, 46]. Global demographic trends suggest that the number of adults aged 60 years or older will double in the coming decades [47] and that the number of patients with several chronic diseases will increase further [43]. Furthermore, to date, there are no evidence-based interventions available to improve personal continuity. Therefore, it is highly warranted to develop strategies to improve continuity of care for older patients.

In this protocol paper, we describe the design of the development and evaluation of a complex intervention for optimising personal continuity for older patients in general practice. Complex interventions are defined as interventions that contain several interacting components and characteristics that should be taken into account such as: behaviour required by those delivering or receiving the intervention, number and variability of outcomes and degree of tailoring of the intervention permitted [48]. Complex interventions are widely used in public health, areas of social policy and health services.

## Methods/design

**Fig. 1:**
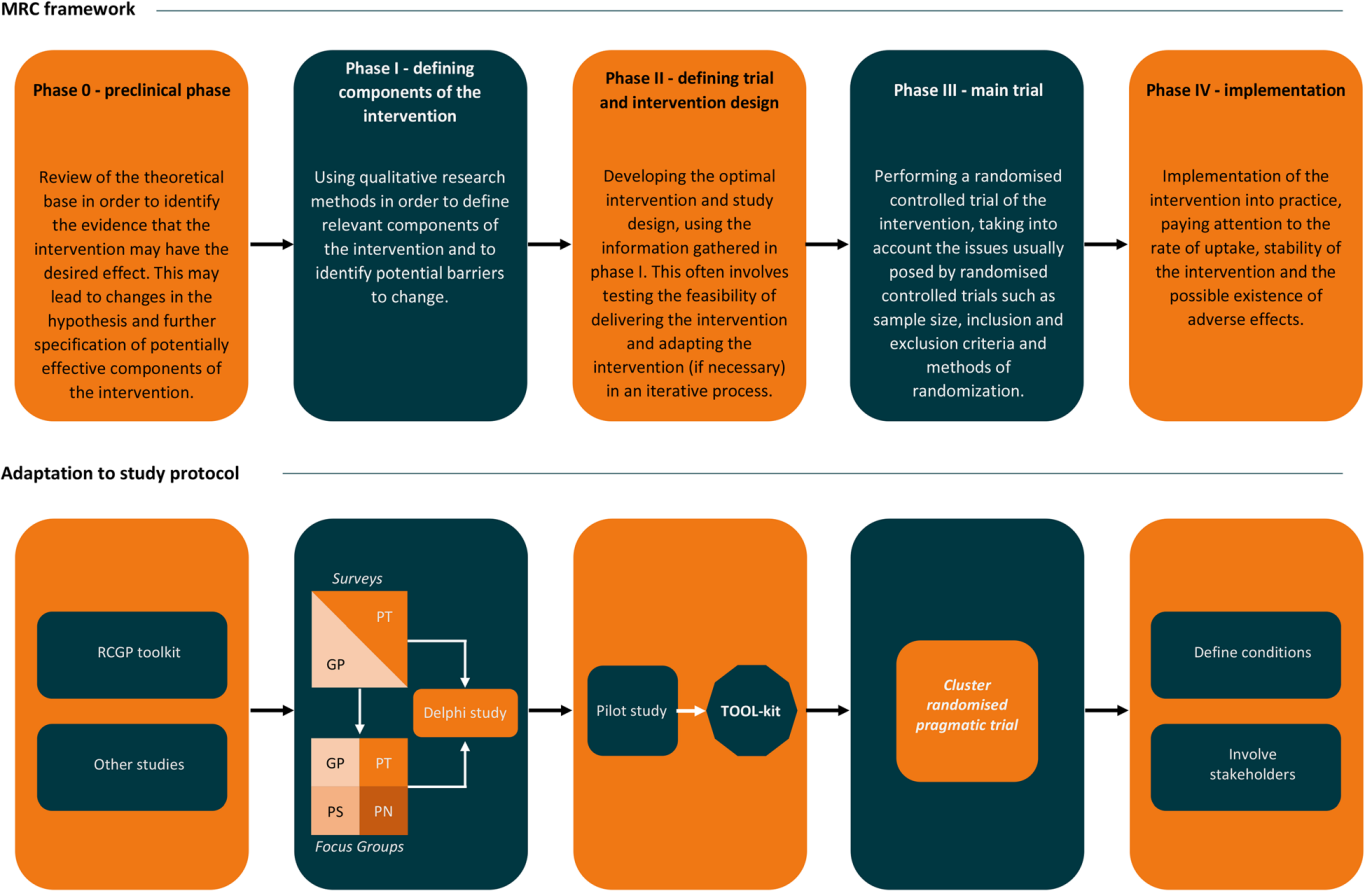
The five phases of the Medical Research Counsil framework for developing and evaluating complex interventions and adaptation to this study. RCGP: Royal College of General Practitioners, GP: General practitioner, PT: patient, PS: Practice Assistant, PN: practice Nurse

The study will consist of five phases where the intervention will first be developed (phase 0, I and II) and subsequently evaluated in a randomised controlled trial (phase III) (Fig. 1). In the last phase, implementation will be assessed (phase IV). The study follows the Medical Research Council (MRC) framework for development and evaluation of complex interventions [48, 49].

### Phase 0: identifying existing evidence

The first step is to identify evidence that the complex intervention may have the desired effect. To this end, the theoretical basis for the intervention will be reviewed using the findings from two members of our research group (HS and AU) and more than 30 international peer-reviewed publications on continuity of care in general practice [1–3, 7, 11, 13–15, 17–35, 46, 50–56]. Secondly, MEDLINE, EMBASE, CINAHL, PsycINFO and Gerolit will be searched to identify evidence on interventions to optimise personal continuity evidence. Thirdly, the Continuity of Care Toolkit developed by the Royal College of General Practitioners (RCGP) in 2014 will be used as a source of potential components for the intervention [57]. The Continuity of Care Toolkit consists of several consensus-based suggestions on how to promote personal continuity in practice (see Additional file 1). Finally, an optimal theoretical basis for the implementation of the intervention will be created using the research findings of Grol and Wensing on successful improving patient care [58].

### Phase I: defining the components of the intervention

Qualitative research methods will be used to determine the relevant components of the intervention and to identify potential barriers to change. Surveys and focus groups will be performed to investigate the views of relevant stakeholders on personal continuity in general practice and how it can be improved. This phase will be concluded by a Delphi study to establish the intervention.

#### GP and patient surveys

Surveys will be used to investigate patients’ and GPs’ views on personal continuity to determine relevant components of the intervention. The surveys will contain questions regarding GPs’ and patients’ views on personal continuity, factors influencing personal continuity and their suggestions on how to improve personal continuity in general practice. Both questionnaires will be based on previous research by Schers et al. [5, 6]. and the results of the literature study performed in phase 0.

For the patient survey, we will recruit 35 GPs from general practices in two regions in the Netherlands and instruct them to take a random sample of 30 patients aged 65 years and older from the electronic medical record (EMR) (*n* = 35*30 = 1050). The selected patients will receive a postal survey with an accompanying letter from their GP. For the GP survey, we will randomly select 100 GPs for participation through various local GP networks. The GPs will receive a digital survey through Survalyzer: an online program for distributing, collecting and analysing surveys [59].

#### Focus groups

In order to prioritise the components of the intervention, focus groups will be organised to discuss the results of the literature study and surveys. GPs, patients, practice nurses and practice assistants will discuss the perceived threats and opportunities for personal continuity in daily practice, share opinions on how to improve personal continuity and discuss perceived barriers and facilitators for introducing these improvements. We aim to include 6–12 participants per focus group and to conduct 4 focus groups of 45–90 min. Two focus groups will consist of only GPs, one will consist of only patients aged 65 years or older, and one will include both practice nurses and practice assistants. Practice assistants are employed in all Dutch general practices [60], perform receptionist duties and have their own consultation hours for supportive medical tasks making them important stakeholders for assessing personal continuity in Dutch general practice. Participants will be purposefully selected in order to maximise the variance in participant characteristics such as gender, age and practice style. An external moderator with experience in moderating focus group discussions and affiliation with the research topic will conduct the focus groups. An observer/recorder will be present at each focus group session. Each focus group will be audio-recorded and transcribed. The transcripts will be checked for accuracy and corrected if necessary.

#### Delphi study

At the end of phase I, the results from the surveys and focus groups will be used to perform a reactive Delphi study among members of our research group (HS, JB, MS, AU, HH and OM) to determine the definite components of the intervention. Here, participants will be asked to give their opinion on predetermined topics in multiple rounds until group consensus is reached [61]. In round one, the members of the research group will receive an overview of draft components from the surveys and focus groups. They will be instructed to rate these draft components on four items: effectiveness and financial, organisational and time feasibility on a five-point Likert scale. Additionally, the members of the research group will be invited to elaborate on their decision and suggest changes. The Likert-scale scores will be trichotomised into negative, neutral and positive scores for purposes of analysis, according to previous research [62, 63]. Consensus will be defined as at least 75% agreement on either a positive, neutral or negative judgement [56]. Next, two members of our research group will independently review the scores of round one and determine which components will be included or excluded from the intervention based on group consensus on the four items.

In round two, the members of the research group will receive an overview of the results of round one, including a list of components on which no consensus has been reached during the review process. For each component on this list, we will ask the members of the research group a single yes/no question (“would you include this component in the intervention”) and invite them to elaborate on their decision. The inclusion or exclusion of components will be determined by majority decision (> 50%). An overview of all components, including scores and comments, will be distributed to the members of our research group inviting them to react to the results.

The literature study, surveys, focus groups and Delphi study will result in a list of definite components which will be conjoined in a complex intervention for optimising personal continuity for older patients: the TOOL-kit.

### Phase II: defining trial and intervention design

A pilot study will be performed to define the trial and finalise the design of the draft TOOL-kit constructed in phase I. Two general practices in two regions of the Netherlands will be included: one practice in the Amsterdam area and one practice in the Nijmegen area. The two practices will be purposefully selected to have different characteristics with regard to practice size, total number of healthcare workers, part-time factor and level of urbanisation. The practices will receive the draft TOOL-kit and will be instructed to review its structure and apply its components to their practice during 2 months. This process will be evaluated using semi-structured interviews with a GP appointed by the practice to examine the acceptability and feasibility of the draft TOOL-kit. The TOOL-kit will be refined and finalised based on the results of the pilot.

### Phase III: evaluation of the intervention and main trial

The constructed TOOL-kit will be evaluated in a stepped wedge, cluster randomised pragmatic trial. In a stepped wedge design, the intervention is rolled-out sequentially to all trial participants over a number of time periods [64]. This design was chosen because we hypothesize that the intervention will do more good than harm and because we do not want to withhold the intervention from a group of participants until the end of the trial duration. Additionally, a stepped-wedge design improves the logistical feasibility of the trial [64] and may contribute to successful inclusion as all participants eventually receive the intervention.

#### Population and sampling

**Fig. 2:**
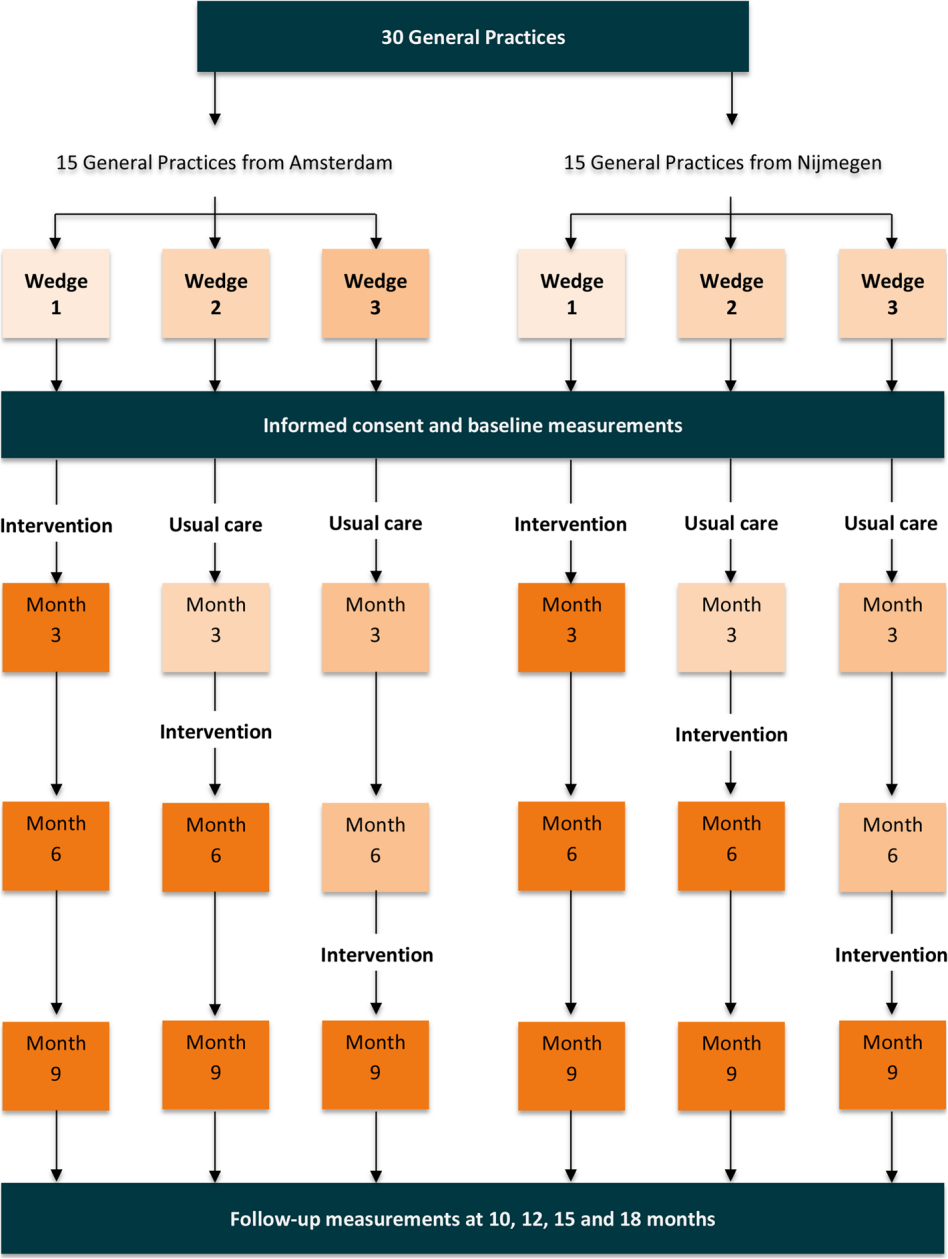
Flowchart of the trial design

30 General practices from the Amsterdam (*n* = 15) and Nijmegen (*n =* 15) areas (Fig. 2) will be included. Practices will be included purposefully to comprise a broad spectrum of practice characteristics based on level of urbanisation, practice size and social-economic status of the practice area. Van Stippend and Schers observed that group practices tend to be more at risk for discontinuity (unpublished results, see Additional file 2). Therefore, only general practices employing three or more GPs will be included.

From the participating general practices, we will recruit GPs and practice assistants for the evaluation of the intervention. All GPs and practice assistants working in permanent employment of the practice are eligible for inclusion. Per practice, a minimum of three GPs and one practice assistant will be included resulting in the enrolment of at least 90 GPs and 30 assistants for the evaluation. This threshold was chosen to comprise a purposeful sample of the study population with sufficient variation in population characteristics while maintaining trial feasibility in regard to recruitment. Participating practices will be asked to bring forward eligible GPs and practice assistants for recruitment at the moment of inclusion.

#### Randomisation and blinding

Practices will be randomised to a wedge using computer-generated random numbers in Microsoft Excel 2016 by a researcher from our group. General practices randomised to wedge 1 will start with the intervention immediately after baseline measurement. Practices of wedge 2 will start in the control condition, providing 3 months of usual care before receiving the intervention. Practices of wedge 3 will start in the control condition, providing usual care for 6 months before start of intervention.

Researchers will be blinded to group assignment during data analysis where possible. Due to the study design and nature of the intervention, researchers, GPs and practice assistants cannot be blinded to group assignment.

#### Informed consent

Individual GPs and practice assistants will receive digital information about the study and a consent form. They can give consent for participation by returning the consent form to a designated researcher from our group.

#### Intervention

The TOOL-kit will be introduced in all participating practices during the course of the study. After receiving the TOOL-kit, practices will be instructed to use it to optimise personal continuity for their older patients. Because the TOOL-kit is introduced on a practice level, all employees and all patients of a practice will be exposed to the intervention. Its introduction is the complex intervention of the study.

The TOOL-kit will consist of multiple components defined in phase 0, I and II. As complex interventions may work best when adapted to local circumstances [48], the TOOL-kit will contain both components which are obligatory and identical for all practices and optional components tailored to the local circumstances of a practice. The components will include professional-oriented strategies (e.g. educational materials, audits and feedback), patient-oriented strategies (e.g. decision aids, arrangements for (direct) contact with healthcare providers) and organisation-oriented strategies (e.g. arrangements for follow-up of patients, revision of professional roles) [58].

#### Usual care

During the trial, all practices will ensure GP care will continue unrestricted. All participating general practices will receive an information leaflet instructing them to continue using the Dutch College of GPs (NHG) guidelines for clinical decision-making during the trial.

#### Outcome measures

The primary outcome measure will be continuity of care from the patients’ perspective using the Nijmegen Continuity Questionnaire (NCQ) (see Table 1). The NCQ consists of 28 items within three subscales: ‘personal continuity: care provider knows me’, ‘personal continuity: care provider shows commitment’ and ‘team/cross-boundary continuity’. Items are scored on a five-point Likert scale, with an additional option to choose ‘?’ (‘I don’t know’). The NCQ has been developed for measuring continuity of care in Dutch general practice and has been validated for measuring and evaluating continuity of care interventions in healthcare systems [65, 66]. As the NCQ is not specifically adapted to the constructed intervention and may not detect all direct or indirect effects, we will perform an additional process evaluation to detect intervention-specific effects [48].

**Table 1:**
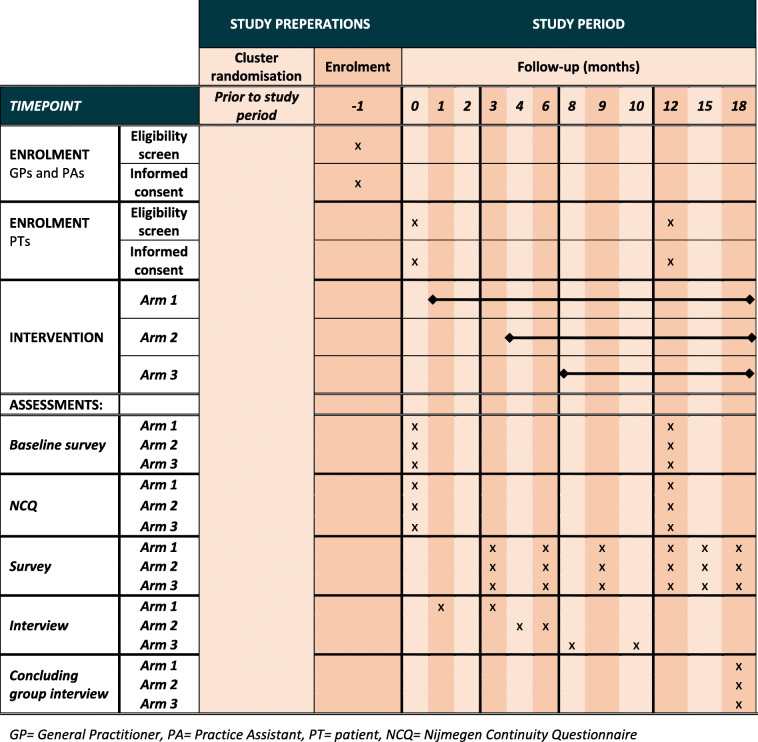
Schedule of enrollment, intervention and follow-up

The secondary outcome measurements during the trial will constitute the process evaluation. This will include measuring implementation, acceptability and feasibility of the TOOL-kit and performing an analysis of barriers and facilitators for successful implementation (Table 1).

Implementation will be evaluated using the guidance proposed by Saunders et al. [67]: the extent to which the components of the TOOL-kit are performed as planned (fidelity), the degree to which the components were provided to GPs and practice assistants (dose delivered) and the extent to which these participants have adopted the components in daily practice and are performing the desired behaviour (dose received).

The acceptability and feasibility of the TOOL-kit will be determined by evaluating the experiences of the GPs and practice assistants with the intervention. This will include the satisfaction with the intervention, its perceived appropriateness to daily practice, work related stress, job satisfaction and support among participants for continued use of the TOOL-kit.

Barriers and facilitators as experienced by the GPs and practice assistants will be collected using a framework proposed by Fleuren et al. (see Additional file 3) [68]. This framework includes 50 factors that can influence the implementation difficulty of a health care innovation.

#### Sample size

The sample size calculation was based on our primary outcome measure (the NCQ). For both NCQ distribution cycles in month 0 and month 12, we will approach 100 patients per practice. We expect at least 35 patients per practice to participate, resulting in a total of 1050 patients in each cycle. When the mean NCQ score is compared between cycles using a mixed linear model with a random effect for practice and two-sided testing at a significance level of 5%, the expected sample size allows detection of all standardized effect sizes of 0.12 and larger with at least 80% power irrespective of the intra-cluster correlation coefficient. The standardized effect size is defined as the difference in means divided by the total standard deviation incorporating both the between- and within-practice variation.

#### Data collection

The NCQ will be distributed to patients in month 0 and 12 in all practices (Table 1). The participating general practices will be provided with a selection algorithm for identification of 100 eligible patients from the EMR. Eligibility criteria are: aged 65 or older, registered at the participating general practice, able to speak Dutch or English, community living, no severe cognitive impairment, and at least 1 GP consultation in the past 12 months. Eligible patients will receive a postal invitation for participation together with the questionnaire.

Digital surveys will be distributed to all GPs and practice assistants using Survalyzer at baseline (month 0) and during follow-up at 3, 6, 9, 12, 15 and 18 months. The baseline survey will address barriers and facilitators for implementation related to the practice organisation, structure and working methods. This survey will contain the same questions for all practices. The other surveys will prospectively measure the implementation, acceptability and feasibility, barriers and facilitators of the intervention during the trial. Because the intervention will be tailor-made per practice, each practice will receive surveys containing questions specifically formulated for that practice to ensure optimal data collection and ability to register unexpected events [48].

Two semi-structured telephone surveys will be held with coordinating GPs of all practices within one month and three months after the introduction of the TOOL-kit in that practice. In interview one, the fidelity, acceptability and other barriers for implementation of the TOOL-kit application will be adressed. Interview two will assess the practice improvement plan for personal continuity on the same parameters as interview one. In month 18, a concluding group interview will be held by videoconference per practice with all GPs and practice assistants to conclude the process evaluation.

The interviews will be conducted by a trained interviewer, voice recorded and transcribed verbatim afterwards.

### Phase IV: implementation

During the final phase, the conditions for large-scale implementation will be defined using the results from phase III. Barriers and facilitators to implementation and potential adverse effects will be determined. For further implementation, local stakeholders and national GP organisations will be involved to integrate the use of the TOOL-kit in daily general practice.

## Analysis

### Surveys (phase I)

The results of our GP survey will be compared to the results of our patient survey using descriptive statistics (sum, mean, standard deviation). Furthermore, the results will be compared to the earlier performed survey studies by Schers et al. [5, 6] using ANOVA and t-test or Kruskall-Wallis ANOVA and Mann-Whitney U tests depending on normality of the data. Associations will be tested using linear regression analysis. All data will be analysed using SPSS (version 26.0).

#### Focus groups (phase I)

Analysis of the transcripts will be conducted by two researchers using the framework method as it allows for a structured approach of the data and easier recognition of patterns [69]. The researchers will first familiarise themselves with the contents of the transcripts and subsequently code parts of the transcript using ATLAS.ti software (v.8.4). The researchers will identify themes using the codes and will review the themes for accuracy in order to identify the relevant theoretical domains.

#### Pilot (phase II)

The semi-structured interviews at the end of the pilot will be performed by a trained interviewer, voice recorded and transcribed verbatim afterwards. Thematic analysis according to the framework method will be performed method as described under *focus groups.*

#### Trial (phase III)

Descriptive statistics will be used to summarize population characteristics. For the primary analysis, the mean NCQ score at baseline and at 12 months follow-up will be compared using a linear mixed model with a random effect for practice and a fixed effect for time point. To analyse if differences depend on longer exposure to the TOOL-kit, the main effect of wedge number and the two-way interaction between wedge number and time point will be determined.

Secondary outcomes measures from the surveys will be compared before and after the intervention and between measurements on different time points using mixed linear models or generalized estimating equations depending on the type of outcome measure. This accounts for clustering of participants within the practices. For outcomes that are collected in more than two periods, the length of exposure to the TOOL-kit main determinant will be considered using the control condition as a reference. A two-sided significance level of 5% will be used for all analyses.

The semi-structured telephone interviews will be analysed according to the framework method described in the *focus groups* section.

## Discussion

This protocol paper describes a multi-phased approach to develop and evaluate a complex intervention for optimising personal continuity for older patients in general practice. Older patients receiving a higher level of continuity of care are likely to be more satisfied and experience a better relationship with their GP [11, 15–17, 23, 53], have a stronger sense of responsibility of their GP [56], a better quality of life [13, 19, 21], less overuse of medical procedures [22], and fewer hospital admissions [11, 23–26]. GPs providing a higher level of personal continuity are more satisfied with their job and perceive to have a better relationship with their patients [15, 16, 18]. In addition, personal continuity improves the uptake of preventive care by patients [11, 14], and increases medication adherence [13].

This study uses the MRC framework to develop and evaluate a complex intervention. The MRC framework was specifically designed to address problems that may arise during the development en evaluation of complex interventions, such as identifying the active ingredient of the intervention and transferability of results [49]. By using the MRC framework, we aim to address these challenges and increase reproducibility of intervention and results.

To our knowledge, this study is the first to investigate an intervention for improving personal continuity in general practice. Therefore, the results of our study will fill an important, previously described, gap of knowledge in respect to interventions for improving personal continuity in general practice [36, 70]. Another strength is the pragmatic nature of the study which enables evaluation of the TOOL-kit as if it would be applied in real-life general practice. This could improve the applicability and external validity of results [71, 72] and may facilitate future implementation [72].

Phase III and IV of the study will probably take place during the current SARS-CoV-2 (coronavirus) pandemic. Because especially complex interventions interact with the context they are performed in, [73] it is likely that the effect of the intervention will be affected by the coronavirus pandemic. We aim to mitigate this effect by dedicating part of the interviews and surveys in our process evaluation to the influence of the coronavirus on general practice. Additional reminders will be sent to reduce non-response and practices will be contacted periodically to assess trial feasibility within that practice and to tailor design adaptions when necessary and where possible.

All phases of the study will focus on personal continuity in Dutch general practice. Although this increases the relevance of the study results for Dutch clinical practice and healthcare policies, additional translational steps may be required to ensure its relevance in other countries.

In summary, the overall aim of this study is to develop and evaluate a complex intervention for improving personal continuity: the TOOL-kit. We will do this by performing a literature study (phase 0), a survey, focus groups and a Delphi study (phase I) followed by a pilot (phase II) and a cluster randomised stepped wedge pragmatic trial (phase III). We will use the results of phase III to define the conditions for further implementation of the TOOL-kit (phase IV).

## Supplementary Information

**Additional file 1.** Royal College of General Practitioners Continuity of Care Toolkit.

**Additional file 2.** Unpublished observations by van Stippend and Schers (2016). The Usual Provider Continuity index (UPC) was calculated among 9 general practices in the Nijmegen area (the Netherlands). The UPC is used to measure personal continuity and is calculated as the number of contacts with the own GP divided by the total number of general practice contacts during the study period. The score of the UPC index varies between 0 (low personal continuity) and 1 (high personal continuity)

**Additional file 3.** Description of the determinants for implementation of healthcare innovations.

**Additional file 4.** Data management plan.

## Data Availability

The dataset(s) supporting the conclusions of this article will be made available in the DataverseNL UBVU after data collection has ended. The details of intended methods of data entry, coding processes and measures to promote data quality and security are described elsewhere (see Additional file 4).

## Abbreviations

EMR: Electronic Medical Record
GP: General practitioner
MRC: Medical Research Council
NCQ: Nijmegen Continuity Questionnaire
NHG: Dutch College of GPs
RCGP: Royal College of General Practitioners
