# Supplementary material for: Optimising personal continuity for older patients in general practice: a study protocol for a cluster randomised stepped wedge pragmatic trial
Source: BMC Fam Pract. 2021 Oct 20;22:207. doi: 10.1186/s12875-021-01511-y (PMC8526277; doi:10.1186/s12875-021-01511-y)
Supplement: Supplementary file 1 — Additional file 1. Royal College of General Practitioners Continuity of Care Toolkit. [file 12875_2021_1511_MOESM1_ESM.pdf]

Supplement 1: Royal College of General Practitioners Continuity of Care Toolkit

Royal College of General Practitioners

RCGP Continuity of Care Toolkit

-Helping clinicians and practices maximise relationship continuity

Introduction

This document considers relationship (interpersonal) continuity – how our systems provide continuity and utilise the therapeutic bond that exists and develops when a patient knows the doctor or nurse well over a period of time. This document also considers the practical steps that can be implemented to support increasing continuity of care within primary care. Recognising that relational continuity provides the foundation for safe, efficient and coordinated care especially for those patients with complex and multiple needs. [*]

Evidence demonstrates that:

1. Continuity of care is important for cost-effective care and patient satisfaction.
2. Maintaining continuity of care is being tested as ways of working within primary care have been changing: larger GP practices, increased numbers of GPs working part-time, the increasing mobility of both staff and patients and the increased pressure on GPs to do more have all contributed to this.
3. As GPs diversify in their roles experienced clinicians are taking on increasing managerial roles which has an impact on the patient facing workforce and impact on continuity of care.
4. There is a tension building between continuity and access of provision. Certain groups of patients place a high value on continuity of care, and often prioritise it over quick access, particularly for chronic, complex and emotional problems. There are some patients however for whim continuity is not as important and being able to access primary care services quickly is paramount. For many patients getting access to appointments though continues to be a challenge.
5. Practices need support to establish how they can improve and utilise continuity of care effectively to improve care for their patients and improve staff satisfaction.

What does the toolkit offer?

We know that when patients can see their chosen clinician this reduces costs (referrals, admissions, operations, and A&E attendance), enables patients to make clearer choices, and improves both patient and staff satisfaction.[3,4] There is also an association between seeing the same doctor and lower mortality rates.[5]

Granier [6] reported that GPs diagnosed meningitis better "when they knew the family." Patients disclose more, GPs get to the point more quickly, both agree things more quickly and patients follow advice and adhere to medication significantly more when there is good continuity. As an example, adherence to statin medication increases by 5% which is associated with a reduction in heart attacks. Higher GP continuity has been associated with lower consultation rates.[4] Seeing patients who are more satisfied on average is easier work and continuity increases GP job satisfaction[7] in addition patients will "forgive" a mistake by their GP if the previous care has been good.[8]

Patients are living longer, and we are realising that the rise of multi-morbidity makes it ever more important for clinicians to know patients well in their context. In the face of these challenges, it is down to individual GPs and practices to reflect on what they can do to enhance continuity for their patients, and this toolkit aims to support that process.

Yet several factors in recent years have made it more difficult for GPs to provide continuity of care. A 2012 study based on the GP Patient Survey showed that while nearly two-thirds of English patients expressed a preference to see a particular GP, a substantial minority of these, up to one-quarter, were, most of the time, not successful in seeing their preferred doctor. [9] And continuity has declined further with the proportion of patients able to see their preferred GP in England falling by 27.5% between 2012 and 2017.[10]

This toolkit focuses on how Practices might look to increase longitudinal continuity, that is where the patient sees the same healthcare professional over time,

It is helpful for practices to review the current level of relationship continuity that is present within their practice. Asking the question around whether patients easily see the doctor they want to when they see this as being a priority?

The Health Foundation and RCGP are working together on a national project over the period 2018 to 2020 looking at ways to improve continuity of care in five centres across England.[17] A range of approaches are being developed in the real life setting of busy general practices. Some centres include all patients, and some are focused on specific groups such as palliative care or those attending frequently.[17]

Steps to improving continuity of care in the modern practice

A. First 'diagnose' the problem

Agree that the practice wants to look at the issue – are people concerned or not?

Then find out the scale of the problem - check what is really happening and collect some data to give you objective evidence.
In England and Scotland, practices can collate and share comparative data from the National GP Patient Survey on whether patients feel they are able to see their preferred doctor.[*] This can be followed by more in-depth work to diagnose the problem such as:

- Finding out how many different people patients are seeing
- Collecting stories about continuity.

TASK 1
Take a sample of patients seen over one year. This could be a random sample of all patients, or perhaps better restricted to frequent attenders – perhaps those attending five times or more over the previous 12 months. For each patient count the number of consultations (N) and the Number of Different Clinicians (NDC) each patient has seen. Ask whether the results are (a) acceptable and (b) desirable?

[*] the 2019 NHS England survey available at http://www.gp-patient.co.uk and questions relating to "having a GP they prefer to see" (overall 52% 2019, 54% 2018) and "almost always see their preferred GP (overall 48% 2019, 50% 2018). For Scotland see https://www2.gov.scot/Topics/Statistics/Browse/Health/GPPatientExperienceSurvey

TASK 2

If possible, also count the proportion of contacts that are with the 'usual doctor'. There are several ways to do this and one widely used approach is the Usual Provider Continuity index (UPC). Very good is around 85%, while some practices average less than 50%; 60% is creditable. Use the* Continuity of Care Calculator† *to tabulate UPC. A two-year data period is advised to ensure enough patient-clinician contacts to calculate stable continuity rates as it overestimates continuity if there are less than three consultations. The UPC identifies the doctor most frequently seen and works with individual doctors, non-doctors, locum and salaried posts.*

The SLICC (St Leonards Index of continuity of care)[2] is an alternative which has advantages over the UPC, but only applies if a personal list with a GP has been fully established across a group of GPs. #

TASK 3

Feed the data back to people: ask specific staff members to seek out some case studies, and to prepare their response to the data.

TASK 4

Discuss the results in a planned meeting with protected time: an away day might be a good place to have these discussions. Flesh out these numbers with examples – stories from both staff and patients - of either, good continuity and its effects, or of bad, with wasted tests, too many prescriptions and delayed diagnosis. At the end, ensure time to decide whether the practice regards this as needing action.

TASK 5

Spread the debate by summarising key concerns, and get suggestions from those at the sharp end, especially patients and receptionists.

TASK 6

If the practice has a patient group *this is a good chance to use their expertise. Have personal continuity as a priority agenda item and supplement this with a comments link on the practice website. Publicise the issue with waiting room displays.*

B. Plan changes to improve continuity

Work with staff and patients to develop suitable policies. The reception team are vital to the success of changing the way the practice approaches relationship continuity. Organise further mini meetings to share views, explain why it matters and to get their detailed suggestions on how to improve.

Having all the practice team on board with the proposed changes is really important before you launch a new way of working.

# The St Leonard's Index of Continuity Care[2] looks at the number of times seen by the named usual doctor compared to other doctors with patient lists. Other examples of measures which are slightly more complex include the Bice-Boxerman Continuity of Care Index, the Herfindahl Index and the Sequential Continuity Index.

* Usual doctor is often the one most consulted in the past 12 months, or else the patient's chosen clinician – these may differ and practices can choose what suits them. Some patients may wish to nominate several!

† UPC is a simple continuity measure of how much patients actually see the same provider (clinician). Of course seeing the same person does not ensure a therapeutic relationship, but adequate continuity is essential to allow relationships to form and flourish.

We are very grateful to Professor Chris Salisbury and Mairead Murphy at the University of Bristol. They have devised a simple Continuity of Care Calculator, available free of charge at http://www.bristol.ac.uk/primaryhealthcare/resources/continuityaudit/

Practicalities – Specifically consider these possible moves:

1. _ Reception and appointments

1.1 Highlight the patient's 'usual doctor*' in the records system and make this doctor the default option for this patient†. Include salaried and part-time clinicians as usual doctors (if contracted for a year or more). Where clinicians have limited availability then a 'first preference' alternate may need to be chosen.

1.2 Encourage patients (and carers when appropriate) to ask for individual doctors and nurses by name. For example, respond to each appointment request with 'who is your usual doctor?'

1.3 Patients should see their usual doctor for medication reviews‡. This reduces the risk of inappropriate prescriptions.

1.4 Also, for test results see/contact either the usual doctor (or perhaps the one who ordered the tests). Again, this will help reduce unnecessary repeat consultations.

1.5 Prioritise supporting patients seeing their usual doctor over ensuring quick access – unless this is not their preference, or it's a pressing emergency and their usual doctor is not available.

1.6 If the patient asks for a very popular or otherwise unavailable doctor, then look for second or third choices.

1.7 If some doctors are requested much more than others, then develop a practice policy for encouraging patients who feel less strongly about who they see to consult with more available doctors.

1.8 Ensure that the practice considers the balance between continuity and access for appointments. Triage systems can sometimes prioritise access and overlook continuity with more capacity reserved for same day appointments. Avoiding restrictions about which GPs are available to handle same day or more urgent requests for contact or appointments can be helpful.

1.9 Try to ensure as far as possible that each GP has appointments spread across the week and at different times of day or is paired up with another part-timer so that between them one is available.

1.10 Explain these policies to new patients and update the practice leaflet. Help receptionists to support the new policies by providing specific training and troubleshooting. Arrange regular feedback sessions.

2. _ Telephone, email and other technologies

As the College's 2019 document 'Fit for the Future document states "There will be an increase in the proportion of consultations that are conducted remotely by video calls, telephone or online. GPs and their practice teams will (need) the time and skills to conduct consultations through a range of communication channels"[16].

Some evidence suggests that initial triage can risk excessive repeat consultations, while telephone follow up with known and trusted clinician saves time and cost.

2.1 Try directing follow up consultations to telephone with a known clinician – normally the usual doctor. Clinicians can explicitly build telephone consultations into follow-up slots.

---

* See previous footnotes – practices can decide how they wish to define 'usual doctor'. Even if most doctors are part time it is still best to allow patients to nominate their preferences – or perhaps their preferred pair or small team - see below.

† This may require system upgrades that are not yet generally available.

‡ Pharmacists can offer vital technical expertise here but cannot replace the all-round vision of the expert medical generalist in prioritising the regime as a whole.

2.2 Email with the usual doctor has potential to save consultations but also could generate unmanageable workload. Medico-legal issues around confidentiality need further work, as do certain practicalities, including implications for patient safety. However, for some patients, contact with their usual doctor or nurse can be extended by using email or other technologies with selected patients and/or within specified regimes for chronic problems – provided both parties are comfortable with this. This is an important area for further development and evaluation.

3. _ In the consulting room

Discuss continuity with patients - emphasise that we can look after them better if they are seeing someone they know but listen to their views and reasons. Key moments are the initial welcome and the end of consultations, especially the management of follow up. Less assertive patients are confused if a doctor whom they had not asked to see invites them to return for follow up and may wonder if it is OK for them to go back to their usual GP or not? So:

3.1 Support the policies operating in reception.

3.2 Explain when it's important for patients to see the same doctor or nurse.

3.3 Avoid confusing patients - do not ask them to return at a time when the appropriate clinician is not available[*].

3.4 Consider introducing repeat appointment slips allowing the clinician to specify either a more or alternatively a less exact interval before a new appointment; this gives receptionists more flexibility to achieve continuity. Alternatively, clinicians can book follow up appointments themselves in negotiation with patients.

3.5 Maximise future access by minimising avoidable follow up. Lengthen intervals for recall and medication review whenever possible and avoid staff shortage times such as holidays and school half term.

3.6 Encourage accessibility by telephone and, if appropriate, by email.

3.7 Consider the review of all non-emergency specialist referrals by the usual doctor.

3.8 Housebound patients should ideally be visited by their usual doctor. Their notes should be flagged as 'housebound' and they merit care plans analogous to those needed for palliative care. While geographic and time constraints can make this difficult for acute visits, consider the potential for avoiding inappropriate and costly tests and admissions.

4. _ Priority groups of patients

Vulnerable patients and those with poor English or lacking the assertive social skills needed may find negotiating the appointment system difficult and so need extra support. This is particularly important for people who are deprived socially or economically, who tend to be sicker, with more complex problems and so need more continuity.

4.1 Try out an explicit policy of prioritising groups of individuals by record tagging. These could be patients with severe or multiple problems, or who seem to use the services in a chaotic way. This will help choice between more and less demanded clinicians.

4.2 Receptionists can use the tags to be alert for strong reasons when seeing the usual doctor might help. In some cases, it may be appropriate, in discussion with the patient, to limit the appointment system to one or more named clinicians. Only a clinician should be able to override this in individual cases (normally after speaking with the patient). These should be recorded, reviewed, and again discussed with the patient.

4.3 Persistent overbooking of individual doctors demands an agreed policy for sharing workload more evenly, such as allocation of new patients to more available clinicians: this will need addressing with the relevant clinician as well as staff and patients.

[*] 'appropriate' can either mean the usual doctor, or the doctor being consulted this time

4.4 Record the patient's first language (if not English) – more useful than ethnicity. If patients need translation facilities such as 'Language Line' or an interpreter their notes must be flagged for appropriate booking of the service and extra consultation time.

5. _ Larger practices

Larger practices can offer a range of expertise and more facilities and choice, together with the size and resources for dedicated chronic disease surveillance programmes. This brings several areas of consideration for relationship continuity.

5.1 The balance between 'usual doctor' and the special skills need to run the long-term care of diabetes, hypertension, asthma or depression needs to be considered.

(a) Where the practice 'specialist' (Dr or Nurse X) in, say, diabetes is not the usual doctor (Dr A), then the potential clash of roles needs to be explicit: *for example,* all the patients of Dr A can be invited for review in a session led by Dr X at a time when Dr A is available. This can integrate Dr A's contextual knowledge with Dr X's special skills.

(b) For the increasing number of patients with multi-morbidity it may be more effective for some regimens to be devised and overseen by the 'specialist' but for overall and personal care to be negotiated and reviewed with their usual doctor.

5.2 Individual clinicians have different styles and ways of working. For example, these may contrast 'fast' clinicians who handle more organic problems with slower consulters who attract (or prefer) complex patients with more severe mental as well as physical problems[18].Groups of clinicians need to recognise the strengths of their members and allocate workload (including appointment length) so as to maximise their effectiveness.

5.3 Clinicians can get overbooked and their colleagues may feel they are seeing more new patients. Continuity and patients suffer. One response is careful monitoring and adjustment of appointments and availability, including a close eye on timekeeping. Clinicians need to be aware of how they work and ensure that they achieve a workable timetable and then conform to this, adjusting as necessary. A more radical response is to move to restrict choice by setting up smaller clinical teams within the group (see next section).

In fact patients with multiple complex problems *must* have more time. This is hard to provide in today's context but the feasibility of funding special extra consulting time for the most complex patients has been well tested in the 'Deep End' project in Glasgow[19].

6. _ Smaller teams

Research has been remarkably consistent in showing that continuity problems start when practices exceed four full time clinicians and/or 8,000 registered patients. It is likely that smaller practices will be challenged by greater numbers of part-time clinicians – as is increasingly common. Researchers have been driven to conclude that the only solution lies in splitting larger groups into smaller teams. These may include two or three part-time doctors as in the RCGP example quoted[*]. This involves:

6.1 The practice negotiates the membership of small teams based on availability and complementarity (often called micro-teams). Team members need to be able to work together, to be able to cover the working week between them and to complement each other's skills. There are also issues about offices and sharing. This is a development of many existing informal 'buddying systems', and of the Gold Standard framework for palliative care where more than one GP is named by the patient.

6.2 Patients are invited to sign up for their team. They can prioritise from the list but need to know that normally they will have to stick with a single team even though their favourite clinicians might be spread over more than one team. Signing up may take several months.

[*] The story on pages 29-30 of the RCGP Policy Paper describes this in more detail (reference 3).

6.3 After, say, six months the scheme goes live and patients are restricted to their chosen team for all except emergency care. Unallocated patients and new patients are assigned to the teams with lightest workload (in rotation).

6.4 The scheme is reviewed regularly and adjusted.

C. Review and adjust

As with all practice changes it is essential to review, adjust/improve and also celebrate.

TASK 1
Monitor feedback from staff and patients and repeat the measures of provider continuity after six months using the same approach as before.

TASK 2
After six months meet with staff and patients. Celebrate any improvements. Discuss changes in provider continuity then revise and improve the new practice policies on continuity. Monitor care processes & outcomes such as prescribing, referrals, tests and quality of care targets.

TASK 3
Feedback progress both to the team and to the patient group and use waiting room notices and practice websites and leaflets. Hopefully there will be evident progress, but problems may also be noted. These may need further specific examination. If many further changes are made, regular review of provider continuity is still helpful.

TASK 4
After one year, review again in full.

TASK 5
Celebrate success and reward those who have played leading roles. Inform the Commissioning Group/Health Board/Primary Care Network about significant progress with an important aspect of primary care. Hopefully you can demonstrate the resulting improved satisfaction ratings on the patient Surveys. Remember these audits can go towards appraisal documents and CQC reports. Any increase in continuity should improve patient care, satisfaction, wellbeing, morbidity and mortality. This should be coupled with reduced admissions, tests and referrals together with greater staff morale!

Updated revision 1st October 2019
Mark Rickenbach (RCGP)
George Freeman (Imperial College)

Original document 2014
George Freeman (Imperial College) for **RCGP Continuity of Care Toolkit Group**:
Saqib Anwar (Leicester & RCGP First5), Alison Dalal (Paddington),
John Duncan (Aberdeen & RCGP Scotland), Hitesh Jiandani (Belfast & RCGP N Ireland),
Bridget Osborne (Conwy & RCGP Wales),
Nigel Watson (New Milton & Wessex LMCs), David Wingfield (Hammersmith),
Amanda Howe (Norwich & RCGP England), Jonathan Ware

References

1 Guthrie B, Saultz J, Freeman GK, Haggerty J. Continuity of care matters: relationships between doctors and patients are central to good care. *BMJ* 2008;**337:**a867 (BMJ Com 7th Aug; printed 6th Sept:548-9).

2 Sidaway-Lee, K., Pereira Gray, D. and Evans, P. (2019.) A method for measuring continuity of care in day-to-day general practice: a quantitative analysis of appointment data *Br J Gen Pract*, **69** (682) :e356-362.

3 Hill AP, Freeman GK. *Promoting Continuity of Care in General Practice*. RCGP Policy Paper, RCGP London March 2011.

4 Barker I, Steventon A, Deely SR. Association between continuity of care in general practice and hospital admissions for ambulatory care sensitive conditions: cross sectional study of routinely collected, person level data. *BMJ* 2017;356:j84 doi:10.1136/bmj.84

5 Pereira Gray DJ, Sidaway-Lee K, White E, Thorne A, Evans PH. Continuity of care with doctors—a matter of life and death? A systematic review of continuity of care and mortality. BMJ Open 2018;0:e021161. doi:10.1136/bmjopen-2017-021161.

6 Granier S, Owen P, Pill R and Jacobsen L (1998) Recognising meningococcal disease in primary care: qualitative study of how general practitioners process clinical and contextual information *BMJ* 1998; **3**:276-9.

7 Ridd, M., Shaw, A. and Salisbury, C (2006) 'Two sides of the coin'—the value of personal continuity to GPs: a qualitative interview study *Family Practice*; **23** (4) :461-468.

8 Lings, P., Evans, P., Seamark, D., Seamark, C., Sweeney, K., Dixon, M. and Pereira Gray, D. (2003) The doctor-patient relationship in US primary care. *JR Soc Med* 2003; **96** (4) :180-184.

9 Aboulghate A, Abel G, Elliott MN, Parker RA, Campbell J, Lyratzopoulos G, Roland M. Do English patients want continuity of care, and do they receive it? *British Journal of General Practice* 2012;**62**:e567-e575(9).

10 Baird B et al *Innovative Models of General Practice*. Kings Fund report June 2018 p19 (quoting from National GP Patient Survey 2017)

11 Baker, R. and Streatfield, J. (1995) What type of general practice do patients prefer? Exploration of practice characteristics influencing patient satisfaction *Br J Gen Pract*; ***45*** (401):654-659.

12 Tammes P, Payne RA, Salisbury C, Chalder M, Purdy S, Morris RW (2019) The impact of a named GP scheme on continuity of care and emergency hospital admission: a cohort study among older patients in England, 2012–2016 *BMJ Open*2019;9:e029103. doi:10.1136

13 Levy M Fleming L Warner JO and Bush A (2019) Paediatric asthma care in the UK: fragmented and fatally fallible *Br J Gen Pract* **69 :**406-407

14 Risi, L., Bhattie, N., Cockman, P., Hall, J., Ovink, E. Macklin, S. & Freeman, G. (2015) Micro-teams for better continuity in Tower Hamlets *Br J Gen* Pract; **65** (639) :536. doi: 10.3399/bjgp15X687025

15 Howe A. *Medical generalism: why expertise in whole person medicine matters*. Royal College of General Practitioners London June 2012:41-63 *download pdf at* http://www.rcgp.org.uk/policy/rcgp-policy-areas/medical-generalism.aspx

16 *Fit for future: a vision for general practice* RCGP May 2019

17 The Health Foundation. Increasing continuity of care in general practice. A programme to fund targeted quality improvement work to increase continuity in GP practices https://www.health.org.uk/funding-and-partnerships/programmes/increasing-continuity-of-care-in-general-practice (accessed 2019)

18 Howie JGR, Heaney D, Maxwell M. Quality, core values and the general practice consultation: issues of definition, measurement and delivery. *Family Practice* 2004;**21:**458-68.

19 Watt G. *GPs at the Deep End - Connecting with General Practice to Improve Public Health.* Report Sept 2011. *download pdf at:* https://www.gla.ac.uk/researchinstitutes/healthwellbeing/research/generalpractice/deepend/ (accessed 2019)
