# Supplementary material for: Optimising personal continuity for older patients in general practice: a study protocol for a cluster randomised stepped wedge pragmatic trial
Source: BMC Fam Pract. 2021 Oct 20;22:207. doi: 10.1186/s12875-021-01511-y (PMC8526277; doi:10.1186/s12875-021-01511-y)
Supplement: Supplementary file 2 — Additional file 2. Unpublished observations by van Stippend and Schers (2016). The Usual Provider Continuity index (UPC) was calculated among 9 general practices in the Nijmegen area (the Netherlands). The UPC is used to measure personal continuity and is calculated as the number of contacts with the own GP divided by the total number of general practice contacts during the study period. The score of the UPC index varies between 0 (low personal continuity) and 1 (high personal continuity) [file 12875_2021_1511_MOESM2_ESM.pdf]

Supplement 2. Unpublished observations by van Stippend and Schers (2016)

Practice type	Number of practices	Average usual provider continuity index	Standard deviation
Single-handed practice	2	0,79	0,15
Two-handed practice	4	0,63	0,04
Group Practice	3	0,56	0,07
**Total**	**9**	**0,64**	**0,11**
