# Supplementary material for: Optimising personal continuity for older patients in general practice: a study protocol for a cluster randomised stepped wedge pragmatic trial
Source: BMC Fam Pract. 2021 Oct 20;22:207. doi: 10.1186/s12875-021-01511-y (PMC8526277; doi:10.1186/s12875-021-01511-y)
Supplement: Supplementary file 3 — Additional file 3. Description of the determinants for implementation of healthcare innovations. [file 12875_2021_1511_MOESM3_ESM.pdf]

Supplement 3. Description of the determinants for implementation of healthcare innovations as described by Fleuren et al.

**Determinants related to the socio-political context**
1. Willingness of the patient to cooperate with the innovation
2. Degree to which the patient is aware of the health benefits of the innovation
3. Patient doubts concerning the health professional's expertise and competence with respect to the
4. Financial burden of the innovation imposed on the patient (e.g. no insurance coverage)
5. Patient discomfort (physical or emotional) as a result of the innovation
6. The extent to which the innovation fits into existing rules, regulations, and legislation
**Determinants related to the organization**
7. Decision-making process and procedures in the organization: top-down or bottom-up/participatory
8. Hierarchical structure: extent to which decision-making process is formalized through hierarchical
9. Formal reinforcement by management to integrate innovation into organizational policies
10. Organizational size (number of employees): large, medium size, small
11. Functional structure (task oriented) versus product structure (output oriented)
12. Relationship with other departments or organizations: introvert or outreaching
13. Nature of the collaboration between departments involved in the innovation
14. Staff turnover: high, average, low
15. Degree of staff capacity in the organization or department that implements the innovation
16. Available expertise, in relation to the innovation in the organization or department
17. Logistical procedures related to the innovation, e.g. logistical problems in scheduling patients
18. Number of potential users to be reached: many, few
**Determinants related to the adopting person/user/health professional**

19. Support from/of colleagues in implementing the innovation
20. Support from/of other health professionals in implementing the innovation
21. Support from/of their supervisors in the department/organization with respect to the implementation
22. Support from/of higher management in the organization with respect to the implementation of the
23. Extent to which colleagues implement the innovation (modelling)
24. Extent to which the health professional has the skills needed to implement the innovation
25. Extent to which the health professional has the knowledge needed to implement the innovation
26. Self-efficacy: confidence to perform the behaviour needed to implement the innovation
27. Extent to which ownership by the health professionals is perceived
28. Extent to which the innovation fits in the perceived task orientation of the health professional
29. Extent to which the health professional expects that the patient will cooperate in the innovation
30. Extent to which the health professional expects that the patient will be satisfied with the innovation
31. Extent to which the health professional suffers from work-related stress
32. Extent to which goals of health professionals with respect to the innovation are contradictory
33. Extent to which the health professional has ethical problems with the innovation
**Determinants related to the innovation**
34. Extent to which the procedures/guidelines of the innovation are clear
35. Compatibility: degree to which the innovation is perceived as consistent with existing work procedures
36. Trialability: extent to which the innovation can be subjected to trial
37. Relative advantage: extent to which the innovation is perceived as advantageous
38. Observability: degree to which the results of the innovations are observable to the health professional
39. Extent to which the innovation is appealing to use
40. Relevance of the innovation for the patient: extent to which the innovation has added value
41. Extent to which the innovation carries risks to the patient compared with the existing situation

42. Frequency of use of the innovation: high, low
**Determinants related to facilities needed to implement the innovation**
43. Financial resources made available for implementing the innovation
44. Reimbursement for health professionals/organizations to facilitate extra efforts in applying the
45. Other resources made available for implementing the innovation (e.g. equipment, manuals)
46. Administrative support available to the users (health professionals) of the implementation
47. Time available to implement the innovation
48. Availability of staff responsible for coordinating implementation in the organization/department
49. Health professionals are involved in the development of the innovation
50. Opinion leader who influences opinions of others in the organization or department (not the

Some determinants, such as reimbursement (number 44), can also be classified in another category, e.g. as a characteristic of the organization.
