# Supplementary material for: Optimising personal continuity for older patients in general practice: a study protocol for a cluster randomised stepped wedge pragmatic trial
Source: BMC Fam Pract. 2021 Oct 20;22:207. doi: 10.1186/s12875-021-01511-y (PMC8526277; doi:10.1186/s12875-021-01511-y)
Supplement: Supplementary file 4 — Additional file 4. Data management plan. [file 12875_2021_1511_MOESM4_ESM.pdf]

Optimising personal continuity for older patients in general practice: a stepped wedge cluster randomised controlled trial

A Data Management Plan created using DMPonline

Creator: Otto Maarsingh

Affiliation: Other

Template: ZonMw (Nederlands)

ORCID iD: 0000-0002-3747-9217

Project abstract:

BACKGROUND Continuity of care denotes the connected and coherent care that is consistent with the health needs and personal circumstances of a patient. It is a widely accepted core principle of general practice. Continuity of care is a multidimensionalconcept, including personal continuity, team continuity, and cross-boundary continuity. This research project aims to optimise personal continuity for older patients in general practice. The benefits of personal continuity have been studied extensively and include a better patient-provider relationship, increased patient and doctor satisfaction, improved uptake of preventive care, higher medication adherence, less overuse of medical procedures, higher quality of patients' life, fewer hospital admissions, and less healthcare costs. Several studies also found personal continuity to be associated with lower mortality. But society and healthcare have changed. Patients and doctors are increasingly mobile and solo practice is becoming rare. The number of patients with chronic diseases rises, and patients increasingly receive care from multiple professionals employed by different organisations. Most general practitioners work part-time and reorganize themselves into large group practices. Other healthcare workers such as the practice nurse have entered general practice. All these changes potentially fragment care and reduce personal continuity. Especially older patients suffer the consequences of fragmentation and discontinuity of care, as they are likely to have multiple chronic conditions and benefit the most from personal continuity. Therefore, strategies to improve continuity of care in older patients are highly warranted, especially because global demographic trends suggest that the number of older persons will double in the next 35 years. If proven effective, such strategies will lead to higher patient-doctor satisfaction, higher quality of patients' life, less overuse of medical procedures, reduced hospitalisation, less healthcare costs, and – on the longer term – lower mortality. MAIN OBJECTIVE The overall aim of this project is to develop and evaluate a multi-component intervention ('toolkit') designed to optimise personal continuity for older patients in Dutch general practice. KEY OBJECTIVES I. To identify and assess the scientific evidence for a multi-component intervention to optimise personal continuity for older patients in general practice. II. To define and prioritise the separate components of the intervention. III. To test the feasibility and acceptability of the intervention and adapt the intervention if necessary. IV. To measure the effectiveness of the intervention on personal continuity for older patients in general practice. V. To investigate the level of implementation of the intervention, the barriers and facilitators to implementation, and the possible existence of adverse effects. METHODS For this research project, we adopted the UK Medical Research Council Complex Interventions framework. This framework provides an iterative phased approach to the development and evaluation of complex interventions, suggesting five phases: During phase 0 (preclinical phase), we will perform a literature study to create the theoretical basis for development and implementation of the intervention. During phase 1 (defining components of the intervention), we will perform surveys and focus groups to investigate patients' and care providers' views on personal continuity, and to define the

components of the intervention. The intervention will consist of a toolkit to optimise personal continuity for older patients in general practice. During phase 2 (defining trial and intervention design), we will perform a pilot study and test the acceptability and feasibility of the constructed toolkit. The results will be used to construct the final version of the toolkit. During phase 3 (main trial), we will investigate the effectiveness of the constructed toolkit in a stepped wedge cluster randomised trial. During phase 4 (implementation), we will use database registrations, practice observations, surveys, and semi-structured interviews to investigate the level of implementation. COLLABORATION For the design and execution of this project, a unique collaboration has been initiated between researchers from the VU University Medical Center, the Radboud University Medical Centre, the Maastricht University, the University of Bristol, and the Netherlands Institute for Health Services Research. DELIVERABLES 1. A Continuity of Care Toolkit for Dutch general practice; 2. A PhD thesis, including at least five international peer-reviewed publications on personal continuity (survey, focus group study, design study, intervention study, and process evaluation study); 3. Patient information sheet on personal continuity, available on www.thuisarts.nl; 4. Position paper ‘Personal continuity’, which could be added to the ‘Toekomstvisie Huisartsenzorg 2022’ (‘Future Vision General Practice Care 2022’).

Last modified: 12-10-2018

Optimising personal continuity for older patients in general practice: a stepped wedge cluster randomised controlled trial - Datamanagement ZonMw

1. Kenmerken van het project en de dataverzameling

1.1 Contactgegevens projectleider

Dr. Otto R. Maarsingh | General practitioner and Senior researcher

Department of General Practice & Elderly Care Medicine

Location VUmc | C382 | Van der Boechorstraat 7, 1081 BT Amsterdam

T: +31(0)204446049 I M: +31(0)612780287 I E: o.maarsingh@vumc.nl

1.2 Ik heb mijn DMP opgesteld in samenwerking met een expert op het gebied van datamanagement. Noem naam, functie, organisatie/afdeling, telefoonnummer, e-mailadres.

- De expert is verbonden aan mijn vakgroep/instituut.

Klaudia Onnasch | Datamanagement Consultant

Divisie VI - Research Support – Datamanagement

Locatie VUmc | MF - H132 / Postbus 7057, 1007 MB Amsterdam

T: 020-44 49827 | k.onnasch@vumc.nl

1.3 Bij het verzamelen van de data voor mijn project ga ik als volgt te werk:

- Nieuwe data genereren

1.4 Ik maak binnen mijn onderzoek gebruik van:

- Een combinatie van kwantitatieve en kwalitatieve data

Zie ook het antwoord op vraag 1.8.

1.5 Ik ga bestaande data hergebruiken en/of koppelen en ik heb toestemming van de data-eigenaar/eigenaren voor het gebruik/ koppelen van zijn/hun data.

- Nee, ik ga geen bestaande data hergebruiken

1.6 Bij het verzamelen van nieuwe data werk ik samen met andere partijen.

- Ja, het verzamelen van nieuwe data doe ik samen met andere onderzoekers, onderzoeksgroepen.

1.7 Het project voer ik uit in een consortium met twee of meer partners. Binnen het consortium zijn duidelijke afspraken gemaakt over datamanagement en intellectueel eigendom. (Denk hierbij ook aan wat het effect is van veranderingen binnen het consortium voor datamanagement en intellectueel eigendom)

- Ja, er zijn (nog) geen duidelijke afspraken gemaakt m.b.t. datamanagement en intellectueel eigendom (leg uit)

Het eerste overleg met de projectgroep, waarbij o.a. afspraken over datamanagement en intellectueel eigendom aan de orde zullen komen, zal waarschijnlijk in het najaar van 2018 plaatsvinden (het onderzoeksproject zelf start per maart 2019). Voor definitieve afspraken m.b.t. intellectueel eigendom zal de onderzoeksgroep zo nodig gebruik maken van de expertise van een bedrijfsjurist.

1.8 Ik kan een inschatting maken van de omvang van de dataverzameling, namelijk het aantal deelnemers of subjects ("n=") van de dataverzameling en de grootte in giga-/terabytes.

- Ja (noem)

Op dit moment is het nog niet mogelijk een precieze inschatting te maken van de omvang van de dataverzameling cq het aantal giga-terabytes, omdat we een complexe interventie gaan ontwikkelen die we vervolgens gaan onderzoeken. Pas gedurende het onderzoeksproject - na fase 0 (literatuuronderzoek), fase 1 (onderzoek middels vragenlijsten en focusgroepen) en fase 2 (pilot onderzoek) - zal er duidelijkheid komen over a) de verschillende componenten van de complexe interventie, b) de desbetreffende uitkomstmaten en c) de omvang van de hieraan gekoppelde dataverzameling.

Desalniettemin kan op basis van het gehanteerde onderzoeksdesign wel een globale inschatting worden gemaakt van de omvang van de dataverzameling:

- Vragenlijsten onderzoek
  - Vragenlijsten naar patiënten (N=1000)
  - Vragenlijsten naar huisartsen (N=50)
  - Vragenlijsten implementatiefase (N=?)
- Focusgroep onderzoek (gesprekken worden opgenomen en vervolgens uitgeschreven)*
  - Focusgroepen (N=4; totale duur is 4x2uur=8uur)
- Interviews (gesprekken worden opgenomen en vervolgens uitgeschreven)*
  - Interviews met dokterassistentes (N=?)
  - Interviews met praktijkondersteuners (N=?)
  - Interviews met huisartsen (N=?)
  - Interviews met patiënten (N=?)
- Kwantitatieve data pilot onderzoek
  - N=2 huisartsenpraktijken
  - N=200 patiënten
  - Registratie van nog nader te bepalen uitkomstmaten
- Kwantitatieve data interventie onderzoek
  - N=30 huisartsenpraktijken
  - N=3000 patiënten
  - Registratie van nog nader te bepalen uitkomstmaten
  - Follow-up periode van 24 maanden met 4-maandelijkse vragenlijsten (0, 4, 8, 12, 16, 20 en 24 maanden)

*: Herleidbare data zullen worden geanonimiseerd dan wel gepseudonimiseerd (zie ook antwoord bij 2.3).

1.9 De volgende eindproducten van het project stel ik beschikbaar voor vervolgonderzoek en verificatie. (licht kort toe)

- Andere producten (noem)
- Software

- Documentatie van het onderzoeksproces, incl dat van alle betrokkenen
- Documentatie over de data
- Syntaxen
- (verschillende versies van) bewerkte data

Toelichting andere producten: gebruikte vragenlijsten zullen beschikbaar worden gesteld voor vervolgonderzoek en verificatie.

1.10 Gedurende het project heb ik voldoende opslaglocaties en -capaciteit en heb ik een back-up van de data beschikbaar. (Geef een korte toelichting.)

- Ja, voor de opslag en back-up van mijn data maak ik gebruik van de standaardvoorzieningen van mijn instituut.

Voor het delen van data met leden van de onderzoeksgroep binnen de organisatie maken we gebruik van een projectmap met beperkt user beheer. Voor het delen van data met leden van de onderzoeksgroep buiten de organisatie maken we gebruik van SURFfilesender of een beveiligde USB.

2. Wet- en regelgeving (incl privacy)

2.1 Ik ga mensgebonden onderzoek doen en ik ben op de hoogte van en mij houd aan de wet- en regelgeving betreffende privacygevoelige data

- Ja, ik ga mensgebonden onderzoek doen en houd mij aan de Algemene Verordening Gegevensbescherming (AVG)

Op dit moment is niet duidelijk of ons onderzoek WMO-plichtig is of niet. Om die reden zal de onderzoeksgroep het project laten toetsen door METC. We verwachten dat het onderzoek niet WMO-plichtig is, omdat de deelnemers in het onderzoek niet aan handelingen worden onderworpen of gedragsregels worden opgelegd.

2.2 Ik ga mensgebonden onderzoek doen en ik heb geregeld dat ik de data verkrijg met (een vorm van) toestemming van de deelnemers.

- Ja, de toestemingsvorm maakt hergebruik van data mogelijk (In de gedragscode Gezondheidsonderzoek wordt voor 'hergebruik' ook wel de term 'nader gebruik' gebruikt)

Bij het genereren van nieuwe persoonsgegevens vraagt de onderzoeker toestemming van de proefpersoon d.m.v. een toestemmingsformulier (informed consent).

2.3 Ik ga mensgebonden onderzoek doen en ik ga privacygevoelige data beschermen tegen datalekken en/of misbruik.

- Ja, ik laat de data pseudonimiseren. (leg uit hoe en door welke instantie dat gebeurt)

Het is de inzet om de data te laten pseudonimiseren, bij voorkeur door een TTP maar anders door de afdeling datamanagement op onze afdeling. Momenteel is in de breedte van het instituut nog niet duidelijk hoe de pseudonimisering bij wetenschappelijk onderzoek precies zal worden uitgevoerd.

De uitwerking van interviews zal worden verricht door onderzoeksassistenten/studenten van het eigen instituut. Zij zullen verplicht worden een geheimhoudingsverklaring te ondertekenen.

2.4 Ik houd me aan het privacyreglement van de organisatie waaraan ik verbonden ben.

- Ja

3. Data vindbaar maken

3.1 De dataverzameling uit mijn project is vindbaar voor vervolgonderzoek. (Let op: Dit is een kerngegeven dat u aan het einde van uw project aan ZonMw door moet geven.)

- Ja, via een online (metadata-)catalogus of webportaal (noem)
- Ja, via de zoekmachine van het archief (repository) waarin hij is opgeslagen (noem)

We zullen gebruik maken van de repository DataverseNL.

We zullen gebruik maken van de portal NARCIS.

3.2 Voor de beschrijving van de dataverzameling gebruik ik een metadataschema.

- Ja, ik kies een metadataschema dat specifiek is voor mijn vakgebied (noem)

We zullen gebruik maken van het metadataschema van het archief van ons instituut. Dit is waarschijnlijk Dublin Core.

3.3 Ik zal gebruik maken van een Persistent Identifier (PI) om duurzaam naar het databestand te verwijzen. (Let op: Dit is een kerngegeven dat u aan het einde van uw project aan ZonMw door moet geven.)

- Ja, de DOI-code

4. Data toegankelijk maken

4.1 Na afloop van het project zullen de data toegankelijk zijn voor verificatie en vervolgonderzoek.

- Ja, na een embargoperiode (leg uit)

De data zullen na een embargoperiode van 3 maanden toegankelijk zijn voor verificatie en vervolgonderzoek. Het is de bedoeling dat de opbrengst van het onderzoek (een toolkit ter bevordering van persoonlijke continuïteit bij ouderen in de huisartsenpraktijk) met hulp van de NHG en LHV online beschikbaar wordt gemaakt voor huisartsen.

4.2 Na afloop van het project wordt het databestand openbaar toegankelijk, zonder aanvullende voorwaarden (open access).

- Nee, ik verbind voorwaarden aan de toegang tot het databestand (restricted access) (leg uit)

Voor gebruik van (een deel van) de data moet een verzoek worden ingediend bij het Data Access Committee (DAC).

4.3 Ik heb gebruiksvoorwaarden beschikbaar waarmee ik de voorwaarden voor toegang tot mijn databestand uitleg na afloop van het project. Geef een link of Persistent Identifier (Let op: Dit is een kerngegeven dat u aan het einde van uw project aan ZonMw door moet geven.)

- Nee, mijn instituut gaat de gebruiksvoorwaarden in samenwerking met een jurist opstellen.

4.4 In de voorwaarden die ik stel aan het gebruik van mijn data (restricted access), heb ik in ieder geval de hieronder aangekruiste punten opgenomen.

- Voorwaarden met betrekking tot dataveiligheid.
- Over de goedkeuring van data-aanvragen zal een stuurgroep, programmacommissie of projectleider beslissen.
- De vergoeding van kosten, bijvoorbeeld voor het verkrijgen van data.
- De periode van toestemming voor gebruik van de dataset.
- De manier waarop de dataset beschikbaar wordt gemaakt.
- Samenwerking bij het gebruik van de dataset, inclusief afspraken over publicaties, auteurschappen.
- Het delen van data voor commerciele doeleinden. Daarbij houd ik rekening met de bepalingen van het staatssteunrecht
- Of de dataset mag worden gekoppeld aan een andere dataset (privacy)

5. Data interoperabel maken (uitwisselbaar, koppelbaar)

5.1 Ik kies een dataformat zodat mijn data leesbaar zijn voor andere onderzoekers en hun computers ('machine actionable').

- Ja (noem)

De volgende - voor andere onderzoekers leesbare - dataformats zullen worden gebruikt: PDF (.pdf), comma-separated values (.csv), PDF/A (.pdf) en syntaxen van SPSS en ATLAS.ti.

5.2 Ik kies een metadatastandaard zodat mijn data gekoppeld kunnen worden aan andere data. (Let op: Dit is een kerngegeven dat u aan het einde van uw project aan ZonMw door moet geven).

- Ja, ik kies een metadatastandaard uit het overzicht van Biosharing (noem)

We zullen gebruik maken van de metadatastandaard SNOMED CT.

5.3 Ik ga mensgebonden onderzoek doen en heb bij de privacybescherming rekening gehouden met hergebruik van de data en eventuele koppeling met andere databestanden.

- Ja, de deelnemers hebben toestemming gegeven voor hergebruik van de data en de data zijn gepseudonimiseerd

Het is bedoeling om - wanneer het onderzoek daadwerkelijk van start is gegaan - de deelnemers om toestemming te vragen voor hergebruik van de data. Het is de inzet om de data te laten pseudonimiseren, bij voorkeur door een TTP maar anders door de afdeling datamanagement op onze afdeling. Momenteel is in de breedte van het instituut nog niet duidelijk hoe de pseudonimisering bij wetenschappelijk onderzoek precies zal worden uitgevoerd.

6. Data herbruikbaar maken en duurzaam opslaan

6.1 Ik zorg voor een goede kwaliteit en documentatie van de data opdat andere onderzoekers ze kunnen interpreteren en gebruiken ('replication package').

- Ik voer kwaliteitscontroles uit op de data opdat ze compleet, correct en consisitent zijn (leg uit)

Er zal worden gedocumenteerd hoe de data tot stand komen. De syntaxen zullen openbaar worden gemaakt
en chronologische worden toegelicht. De interviewers / onderzoeksassistenten / studenten zullen training en begeleiding ontvangen op het gebied van dataregistratie en -invoer. Er zal zoveel mogelijk gebruik worden gemaakt van vaste data-invoerders. Voor de verwerking van kwalitatieve data zullen we gebruik maken van het programma Atlas Ti.

Daarnaast zal het onderzoeksvoorstel ter goedkeuring aan de METC worden voorgelegd, vinden op onze afdeling terugkerend audits plaats en houdt de promovendus vanaf het begin van het onderzoek een logboek bij.

6.2 Ik heb selectiecriteria om aan het eind van het project te bepalen welk deel van de data moet worden bewaard. (zie uw antwoord op vraag 1.9)

- Nog niet

We zullen in de aanloop naar de start van het onderzoeksproject selectiecriteria formuleren wat betreft het al dan niet bewaren van data.

Verder is nu al besloten om audio data (opbrengst interviews en focusgroepen) na verwerking te vernietigen vanuit het oogpunt van herleidbaarheid en privacy.

6.3 Aan het einde het project kan ik, na de selectie van de data, een inschatting maken van de omvang van het databestand (in Gb/Tb) dat ik voor lange termijn ga opslaan/ archiveren.

- Ja (noem)

Omdat het onderzoeksproject naar verwachting geen grote databestanden zal opleveren (geen volumineuze data zoals bijvoorbeeld MRI data), verwachten we dat opslag op de lange termijn geen problemen zal opleveren cq zal leiden tot extra kosten.

6.4 Ik zorg dat ik aan het eind van het project een keuze heb gemaakt voor een archief of repository voor duurzame lange termijnarchivering (gecertificeerd) van mijn databestand. (Let op: Dit is een kerngegeven dat u aan het einde van uw project aan ZonMw door moet geven.)

- Ja, het archief heeft een data seal of approval (noem het archief)

Repository: DataverseNL UBVU.

6.5 Na afloop van het project, zal ik voor mijn data de aanbevolen bewaartermijn van minimaal 10 jaar hanteren.

- ja volgens de VNSU richtlijnen (noem aantal jaren)

Bewaartermijn: minimaal 10 jaar.

6.6 De kosten voor datamanagement gedurende het project en de voorbereiding van archivering mogen op de projectbegroting. De kosten zijn:

- Onbekend (leg uit)

In afwachting van een definitieve beslissing aangaande de pseudonimisering is dit nu nog niet bekend. De kosten van de opslag an sich zullen - gegeven het verwachte lage aantal Gb en de richtprijs van €4,50 per Gb - verwaarloosbaar klein zijn.

6.7 De kosten van lange termijn archivering van de dataset, na afloop van het project, zijn gedekt.

- Ja (leg uit)

Zie antwoord vraag 6.6.

Optimising personal continuity for older patients in general practice: a stepped wedge cluster randomised controlled trial - Data management ZonMw (English version)

1. General features of the project and data collection

1.1 Project leader contact details

1.2 I have composed my DMP with the assistance of a data management expert. List his or her name, function, organisation/department, phone number and email address.

1.3 In collecting data for my project, I will do the following:

1.4 In my research, I will use:

1.5 I will be reusing or combining existing data, and I have the owner's permission for using or combining their data.

1.6 In collecting new data, I will be collaborating with other parties.

1.7 I am a member of a consortium of 2 or more partners. Clear arrangements have been made regarding data management and intellectual property. (also consider the possible effect of changes within the consortium on issues of data management and intellectual property)

1.8 I can give an estimate of the size of the data collection; specifically, the number of participants or subjects ("n=") in the collection and its size in GB/TB

1.9 The following end products I will make available for further research and verification (please elaborate briefly)

1.10 During the project, I will have access to sufficient storage capacity and sites, and a backup of my data will be available. (please elaborate briefly)

2. Legislation (including privacy)

2.1 I will be doing research involving human subjects, and I am aware of and compliant with laws and regulations concerning privacy sensitive data.

2.2 I will be doing research involving human subjects, and I have (a form of) informed consent from the participants for collecting their data.

2.3 I will be doing research involving human subjects, and I will protect my data against misuse.

2.4 I will stick to the privacy regulations of my organisation

3. Making data findable

3.1 The data collection of my project will be findable for subsequent research (note: this is a key item, which you should report to ZonMw at the end of your project).

3.2 I will use a metadata scheme for the description of my data collection.

3.3 I will be using a persistent identifier as a permanent link to my data collection (note: this is a key item, which you should report to ZonMw at the conclusion of your project).

4. Making data accessible

4.1 Once the project has ended, my data will be accessible for further research and verification.

4.2 Once the project has ended, my data collection will be publicly accessible, without any restrictions (open access).

4.3 I have a set of terms of use available to me, which I will use to define the requirements of access to my data collection once the project has ended (please provide a link or persistent identifier; also note that this is a key item, which you should report to ZonMw at the conclusion of your project).

4.4 In the terms of use restricting access to my data, I have included at least the following:

5. Making data interoperable

5.1 I will select a machine actionable data format, which will allow other researchers and their computers to read my data collection.

5.2 I will select a metadata standard to allow my data collection to be linked to other collections (note: this is a key item, which you should report to ZonMw at the conclusion of your project).

5.3 I will be doing research involving human subjects, and I have taken into account the reuse of data and the potential combination with other data sets when taking privacy protection measurements.

6. Making data reusable

6.1 I will ensure that the data and their documentation will be of sufficient quality to allow other researchers to interpret and reuse them (in a replication package).

6.2 I have a number of selection criteria, which will allow me to determine which part of the data should be preserved once the project has ended. (see also question 1.9)

6.3 Once the project has ended and the data has been selected, I can make an estimate of the size of the data collection (in GB/TB) to be preserved for long-term storage or archival.

6.4 I will select an archive or repository for (certified) long-term archiving of my data collection once the project has ended. (note: this is a key item, which you should report to ZonMw at the conclusion of your project)

6.5 Once the project has ended, I will uphold the recommended data preservation period of at least 10 years.

6.6 Data management costs during the project and preparations for archival can be included in the project budget. These costs are:

6.7 The costs of archiving the data set once the project has ended are covered.
